# Identification and experimental validation of aging-related biomarkers in intervertebral disc degeneration

**DOI:** 10.1038/s41598-026-47889-6

**Published:** 2026-04-09

**Authors:** Fan Zhang, Lei Yuan, Heng Ding, Zhenkai Lou, Xingguo Li

**Affiliations:** Department of Orthopedics, The First Affiliated Hospital of Kunming Medical University, No. 295, Xichang Road, Wuhua District, Kunming, 650032 Yunnan China

**Keywords:** Intervertebral disc degeneration, PPM1D, PIK3C2A, BTG3, Biomarkers, Cell biology, Computational biology and bioinformatics, Diseases, Molecular biology

## Abstract

Intervertebral disc degeneration (IDD) is a prevalent disease with an increasing incidence, and aging is a key risk factor for its progression. Therefore, this study aimed to explore the role of aging-related genes in IDD through bioinformatics analysis. Differentially expressed aging-related genes were obtained from the CellAge, GSE150408 and GSE124272 datasets, followed by biomarker screening via machine learning. Finally, the identified biomarkers were validated using reverse transcription-quantitative polymerase chain reaction (RT-qPCR), Western Blot (WB), and immunohistochemistry (IHC) assays. A total of 33 aging-related differentially expressed genes (DEGs) were screened in IDD, and machine learning combined with ROC curve analysis identified PPM1D, PIK3C2A, and BTG3 as aging-related biomarkers for IDD; Gene Set Enrichment Analysis (GSEA) revealed that these three biomarkers were enriched in gene functions including cellular senescence, multicellular organismal aging, negative regulation of cellular senescence, and Ribosome. In addition, the multifactorial regulatory network showed that transcription factor E2F1 and hsa-miR-147 co-regulated both PPM1D and BTG3. In experiments validating biomarker expression levels, BTG3 and PIK3C2A exhibited consistent expression trends across IHC, RT-qPCR, WB assays and the two datasets. BTG3 and PIK3C2A may play more critical roles in the progression of IDD, thereby providing novel insights for the development of new therapeutic strategies for IDD patients.

**Supplementary Information:**

The online version contains supplementary material available at 10.1038/s41598-026-47889-6.

## Introduction

Low back pain (LBP) is a common disorder, and the elements comprising the lumbar spine are subjected to stressors that often trigger LBP, inflicting physical discomfort on patients and posing a significant economic burden on society^1^. One of the leading causes of LBP is intervertebral disc degeneration (IDD), which is characterized by structural damage, apoptosis of nucleus pulposus cells, release of pro-inflammatory cytokines, and degradation of the extracellular matrix in the intervertebral disc^2^. Previous studies have identified trauma, heritability, and repetitive overloading as the main risk factors for IDD^3^. Aging plays a crucial role in the pathogenesis of IDD. According to a recent study, individuals aged 65 years or older were the second most frequent age group seeking medical attention for LBP. Previous research has shown that the prevalence of LBP increases steadily from adolescence to 60 years of age^4^. However, current treatments for IDD are predominantly focused on relieving pain and delaying the degenerative process, with a lack of highly effective therapeutic strategies. Therefore, there is an urgent need to explore the pathogenesis of IDD to provide a theoretical basis for the development of novel therapeutic regimens.

Aging is defined as a state of progressive functional decline accompanied by an increase in mortality, encompassing impaired regeneration, functional disorders, and structural changes^5^. The chronic accumulation of senescent cells is generally recognized to be closely associated with IDD in the aging population. Histological studies have demonstrated that starting from the second decade of life, the vitality and function of intervertebral disc cells gradually diminish due to reduced blood supply to the vertebral bodies, which contributes to the development of IDD^6^. Furthermore, the senescence and apoptosis of nucleus pulposus cells (NPCs) represent key pathological drivers of IDD^7^. Studies have demonstrated that the overexpression of transcription factor p300 promotes the proliferation and autophagy of NPCs, while concomitantly inhibiting cellular senescence and apoptosis, a biological process linked to the upregulation of FOXO3. Mechanistically, p300 enhances FOXO3 expression by binding to the Sirt1 promoter, which facilitates the inactivation of the Wnt/β-catenin signaling pathway and thereby attenuates the progression of IDD^8^. Collectively, these findings indicate that cellular senescence and apoptosis are closely correlated with the advancement of IDD. Nevertheless, the senescence-associated biomarkers involved in IDD remain to be fully elucidated.

Hence, we performed bioinformatics analysis and experimental validation to identify valuable biomarkers. By examining the correlation between aging-related genes and the development of IDD, new biomarkers may be identified for the diagnosis and personalized treatment of IDD.

## Data and methods

### Raw data collection

In this study, blood transcriptome datasets (GSE150408 and GSE124272) derived from intervertebral disc degeneration (IDD) patients and normal control samples were downloaded from the Gene Expression Omnibus (GEO) database (https://www.ncbi.nlm.nih.gov/geo/browse/?view=series&type=1). Specifically, GSE150408 was designated as the training set, comprising 17 IDD samples and 17 matched normal samples, while GSE124272 served as the validation set, consisting of 8 IDD samples and 8 normal control samples. Both datasets were generated on the GPL21185 sequencing platform and were sourced exclusively from human blood samples. Additionally, aging-related genes were retrieved from the CellAge database (https://ngdc.cncb.ac.cn/databasecommons/database/id/6963), with a total of 279 genes identified as key regulators of cellular senescence.

### Screening of differentially expressed senescence-related genes (DESRGs)

Principal component analysis (PCA) was employed for sample quality assessment of the GSE150408 dataset in this study. Subsequently, we screened differentially expressed genes (DEGs) between normal and IDD samples in GSE150408 (|log_2_FC| >1, adjusted P-value [adj.p.val.] < 0.05), using the R limma package (version 3.46.0)^9^. Furthermore, we used the pheatmap (version 1.0.12) and ggplot2 R packages (version 3.3.4)^10^ to create heat and volcano maps of the DEGs, respectively. The intersection of DEGs and aging-related genes yields aging-associated (DEG s) specific to IDD. A Venn diagram was generated using the online tool Venny (http://bioinfogp.cnb.csic.es/tools/venny/). Furthermore, a heat map of the Spearman correlation analysis for the DESRGs was constructed.

### Functional enrichment analyses and construction of protein-protein interaction network

Gene Ontology (GO) and Kyoto Encyclopedia of Genes and Genomes (KEGG) term enrichment were analyzed using the R package “clusterProfiler” (version 3.18.0)^11^. The bar plots were generated using enrichPlot (version 1.10.2) for the visualization of enrichment analysis results. GO terms included biological processes (BPs), cellular components, molecular functions, and KEGG pathways. Statistically significant results were indicated by an adjusted *P*-value < 0.05. We conducted the STRING database (https://cn.string-db.org/) to construct the protein-protein interaction (PPI) network (confidence = 0.15). The PPI network was visualized using the Cytoscape software (version 3.8.2).

### Acquisition and verification of hub genes

Intersections were used to select hub genes using Least absolute shrinkage and selection operator (LASSO) logistic regression and support vector machine recursive feature elimination (SVM-RFE). In addition, receiver operating characteristic (ROC) curve analysis was conducted using the pROC package (version 1.17.0.1)^12^ to assess the diagnostic reliability of the genes and calculate the area under the curve (AUC), which was used to evaluate the diagnostic reliability of the hub gene characteristics. Hub genes with AUC > 0.7 were considered valid for disease diagnosis. The diagnostic value of genes from the GSE124272 dataset was further verified. Genes with diagnostic significance in both datasets were selected as diagnostic genes.

### Nomogram construction and validation

Based on the identified biomarkers, a nomogram was constructed using the rms R package in the training set samples with available survival data, and calibration curves were plotted via the regplot R package (version 4.3.3). Additionally, decision curves and clinical impact curves were generated using the “rmda” package (version 4.3.3)^13^ to evaluate the clinical value and diagnostic efficacy of these biomarkers.

### Gene set enrichment analysis

We clarified the potential function of monogenic gene set enrichment analysis (GSEA) using the R clusterProfiler package (version 3.18.0) based on the GO-BP and KEGG databases^14,15^. Statistical significance was set at *P* < 0.05.

### Immune infiltration analysis

In this study, the CIBERSORT algorithm (version 1.03)^16^ and the LM22 gene set were used to calculate the proportions of 22 types of immune cells in the blood samples from the GSE150408 dataset. The Mann–Whitney U test (rank-sum test) was applied to compare differences in immune cell proportions between the control group and the IDD group, and box plots were generated for visualization using the ggplot2 package (version 3.3.6) in R software.

### Multifactorial network of biomarkers and drug-gene network analysis

Correlation relationship pairs between the biomarkers and other differential aging-related genes were extracted, the co-expression relationship was screened (*P* < 0.05), and the results were imported into Cytoscape^17^ to establish a network model. NetworkAnalyst (https://www.networkanalyst.ca/)^18^ was used to predict the upstream transcription factors. Possible miRNAs targeting the diagnostic genes were identified from the miRNet (https://www.mirnet.ca/)^19^ online databases. Subsequently, Cytoscape software was used to visualize the results. Gene names were used to identify small-molecule drugs at https://clue.io/. A series of pairs of drug-gene interactions were obtained. Similarly, Cytoscape was used to visualize the networks of gene-drug interaction.

### Clinical sample collection and ethical approval

Lumbar IDD was classified according to the Pfirrmann classification criteria^20^, where Grades I and II were defined as mild IDD and Grades III, IV, and V as severe IDD; nucleus pulposus tissues were obtained from 5 patients with mild IDD and 5 patients with severe IDD who were treated at the Department of Orthopedics, The First Affiliated Hospital of Kunming Medical University. The inclusion criteria included patients without other systemic diseases or severe physical disorders, while the exclusion criteria covered patients with severe physical conditions such as yellow ligament calcification, posterior longitudinal ligament calcification, lumbar tuberculosis, and tumors; based on these criteria, a total of 10 patients were enrolled, with a mean age of < 45 years, including 7 males and 3 females. All patients were informed about the experiment before the collection of nucleus pulposus specimens and signed written informed consent forms, and the study was approved by the Ethics Committee (Approval No.: (2023) Len Audit L No. 176).

### Experimental validation of immunohistochemistry

After sampling, the nucleus pulposus and annulus fibrosus were separated under a dissecting microscope for subsequent experiments. For immunohistochemical analysis, the tissues were fixed in paraformaldehyde, followed by dehydration, clearing, and embedding in paraffin; the paraffin-embedded tissues were sectioned, and the sections were placed in an oven at 60–65 °C for 1–1.5 h and then dewaxed with xylene. The dewaxed tissue sections were rinsed with tap water and soaked in distilled water five times, after which antigen retrieval was performed using a retrieval solution under high temperature and high pressure. The tissue sections were blocked, primary antibodies were added and incubated at 37 °C for 1 h, secondary antibodies were then added and incubated at 37 °C for 30 min, and finally DAB solution was added. The sections were counterstained with hematoxylin, rinsed with tap water for 1–2 min, dehydrated, cleared with xylene, and mounted. The staining of each group was evaluated based on the proportion of strongly positive immunohistochemical staining. The primary antibodies used in this study were as follows: PPM1D antibody (ab2344391, ABCAM, dilution ratio 1:50), BTG3 antibody (PA5-50203, ThermoFisher, dilution ratio 1:100), PIK3C2A antibody (MA5-26506, ThermoFisher, dilution ratio 1:150).

### Validation of expression levels by RT-qPCR

Intervertebral disc tissues were taken on ice for nucleus pulposus tissue isolation and cut into pieces, and total cellular RNA was extracted using a one-step method with TRizol reagent. The total RNA was reversely transcribed into cDNA using a cDNA synthesis kit. RT-qPCR was used to detect the expression levels of PPM1D, BTG3, and PIK3C2A in nucleus pulposus tissues. The RNA sequences used for sequencing of PPM1D, BTG3, and PIK3C2A are provided in Supplementary Table 1. GAPDH was used as the internal reference. The conditions of PCR were as follows: 95 °C for 10 min, 95 °C for 15 s, and 60 °C for 1 min, 40 cycles. In this study, each sample was analyzed in three biological replicates, with three technical replicates per biological replicate. Relative gene expression levels were calculated using the 2^−ΔΔCt^ method, and statistical analyses were performed using GraphPad Software.

### Validation of expression levels by western blot (WB)

Total proteins were extracted from cells using a protein extraction kit (Proteintech, Wuhan, China), and their concentrations were determined via the bicinchoninic acid (BCA) assay. The proteins were transferred onto polyvinylidene fluoride (PVDF) membranes, which were blocked with phosphate-buffered saline (PBS) containing 5% non-fat milk for 1 h at room temperature. Subsequently, the membranes were incubated overnight at 4 °C with specific primary antibodies against the target proteins and GAPDH antibody (used as the internal reference). After three washes with PBST buffer, the membranes were incubated with the corresponding secondary antibodies at 37 °C for 2 h. Following another three rounds of washing with PBST, enhanced chemiluminescence (ECL) detection was performed, and the images were captured using a protein gel imaging system. ImageJ software was applied to analyze the experimental data; GAPDH was used as the internal control for quantifying the relative expression levels of the target proteins. Primary antibodies targeting PPM1D, BTG3 and PIK3C2A were purchased from Abcam (Cambridge, UK).

### Statistical analysis

Statistical analyses for data of our experiments were performed with the Prism software (GraphPad Software, La Jolla, CA). Independent-sample t-test was used to validate significant differences; ∗∗∗ represents *P* < 0.001, ∗∗ represents *P* < 0.01, and ∗ represents *P* < 0.05.

## Results

### Screening of differentially expressed senescence-related genes (DESRGs)

**Fig. 1:**
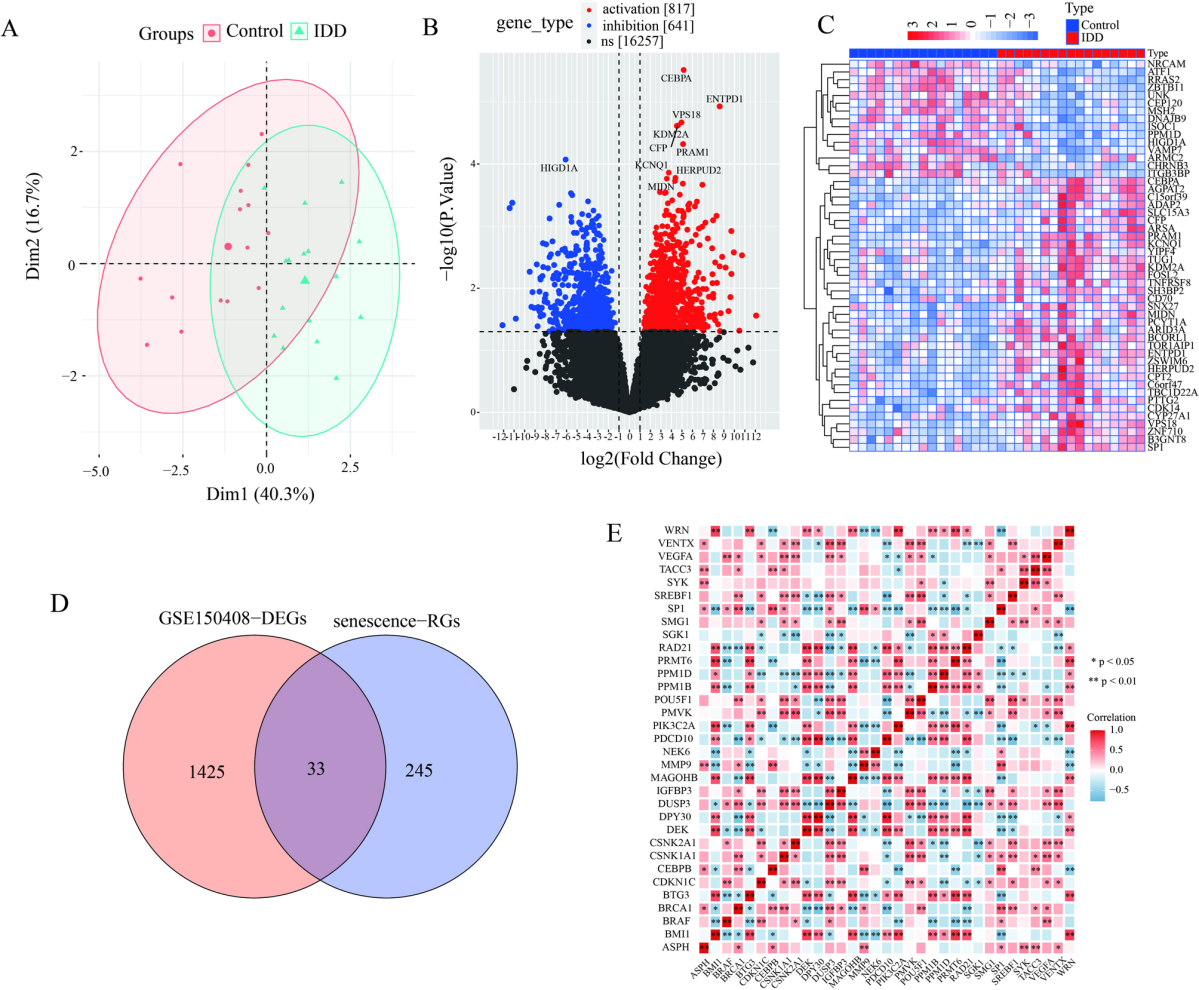
Screening of differentially expressed senescence-related genes (DESRGs). (**A**) PCA Analysis of the GSE150408 dataset. (**B**) Volcano plot of IDD-related DEGs in the GSE150408 dataset. (**C**) Heatmap showing the top 50 IDD-associated DEGs from the GSE150408 dataset. (**D**) Venn diagram illustrating the intersection between DEGs and aging-related genes. (**E**) Correlation heatmap of DESRGs.

In this study, principal component analysis (PCA) was performed to assess the quality of samples in the GSE150408 dataset. Results showed that control and IDD samples were distinctly separated along Dim 1, which accounted for more than 30% of the total variance, indicating a significant difference in gene expression levels between the control and IDD groups (Fig. 1A). The differential analysis results in GSE150408 showed that there were a total of 1458 DEGs between the degenerative and control groups (817 upregulated and 641 downregulated) (Fig. 1B), and the TOP50-DEGs are shown in heatmaps (Fig. 1C). The intersection of 1,458 DEGs and aging-related genes from the CellAge dataset was obtained, yielding a total of 33 overlapping genes. These genes were designated as DESRGs. (Fig. 1D). And correlation analysis was performed between aging-related DEGs in IDD (Fig. 1E).

### Function enrichment of differentially expressed senescence-related genes

**Fig. 2:**
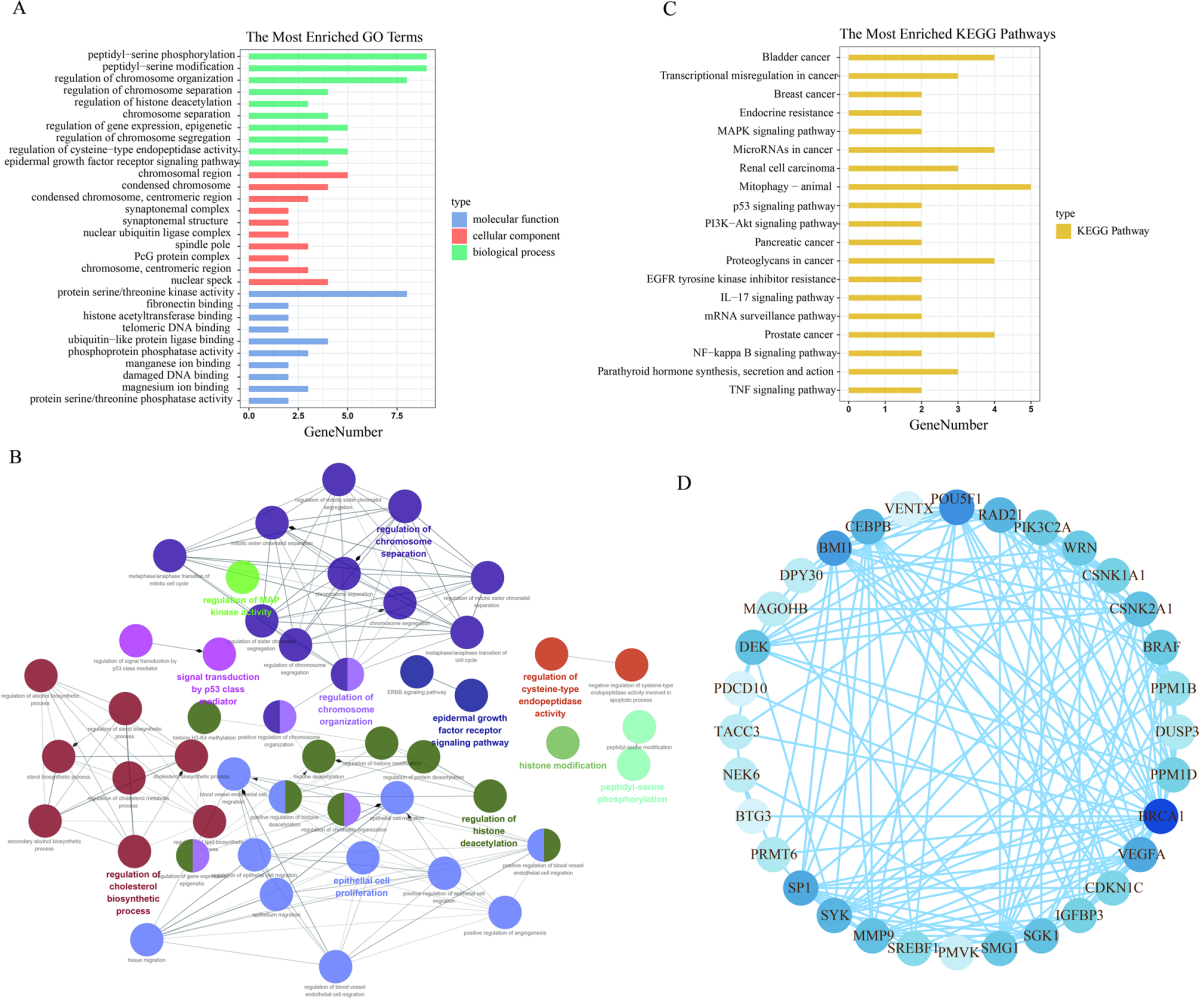
The function enrichment of DESRGs. (**A**) Bar chart of DESRGs’ GO enrichment analysis. (**B**) GO functional enrichment diagram of TOP50 DESRGs. (**C**) A total of 19 pathways were identified by KEGG enrichment. (**D**) PPI network showed interaction relationships between DESRGs.

Functional enrichment analysis was conducted to further elucidate the biological functions of DESRGs. GO enrichment analysis showed that DESRGs were mainly associated with the epidermal growth factor signaling pathway and the regulation of cysteine-type endopeptidase activityin terms of BP. Epidermal growth factor delays the accumulation of lipofuscin-enriched aging pigments in intestinal cells. In terms of cell composition, the DESRGs were associated with nuclear speckles, chromosomes, and mitotic zones. In terms of molecular function, they were related to protein serine/threonine phosphatase activity and magnesium ion binding (Fig. 2A). ClueGO was performed to visualize the link between TOP50 biological functions, which included the regulation of mitogen-activated protein (MAP) kinase activity, epithelial cell proliferation, signal transduction by p53 mediator, and regulation of cholesterol biosynthetic process (Fig. 2B). The KEGG enrichment analysis showed that DESRGs were mainly associated with the tumor necrosis factor (TNF) signaling pathway, parathyroid hormone synthesis, nuclear factor-kappa B signaling pathway, and so on (Fig. 2C). Activating the nuclear factor-kappa B signaling pathway and increasing TNF-α could accelerate aging with higher levels of reactive oxygen species. Finally, Analysis of the PPI regulatory network revealed interactions among 32 proteins (Fig. 2D).

### Screening of biomarkers

**Fig. 3:**
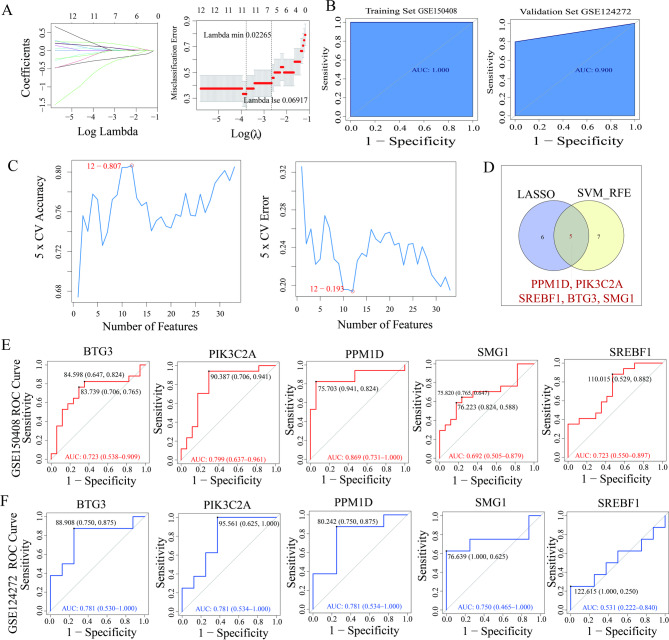
Screening of hub genes. (**A**) Characteristic genes were screened by LASSO regression analysis. (**B**) ROC curve of LASSO regression analysis. (**C**) Characteristic genes were obtained from the SVM feature number, error rate, and precision rate graph. (**D**) Hub genes were identified after the intersection on the Venn diagram (**E**,**F**) ROC curves of hub genes in the training set (GSE150408) and validation set (GSE124272), respectively.

To identify aging-related biomarkers for IDD, this study conducted LASSO and SVM-RFE analyses on 33 intersecting differential genes. When Lambda.min was 0.02265, the penalty coefficient of the genes reached 0, corresponding to 11 characteristic genes (PPM1D, PIK3C2A, CSNK1A1, TACC3, MMP9, CDKN1C, SREBF1, BTG3, SMG1, SGK1, and PRMT6) (Fig. 3A). Meanwhile, the ROC curves of the training set (AUC = 1.0) and validation set (AUC = 0.9) indicated that these 11 characteristic genes had good diagnostic performance (Fig. 3B). Under the SVM-RFE algorithm, when the number of genes varied from 1 to 33, the error rate at the optimal point for predicting disease samples and normal samples was 0.193, with a precision of 0.807; at this point, 12 characteristic genes were obtained (PIK3C2A, CEBPB, PPM1D, SMG1, ASPH, BRCA1, BTG3, CSNK2A1, NEK6, DPY30, DUSP3, and SREBF1) (Fig. 3C). Taking the intersection of the characteristic genes from LASSO and SVM-RFE, a total of 5 hub genes were acquired (PPM1D, PIK3C2A, SREBF1, BTG3, and SMG1) (Fig. 3D). Finally, ROC curves of these 5 hub genes were plotted in both the training set and validation set, and the hub genes with an AUC value greater than 0.7 were identified as the biomarkers for IDD (PPM1D, PIK3C2A, and BTG3) (Fig. 3E,F).

### Clinical application of biomarkers

**Fig. 4:**
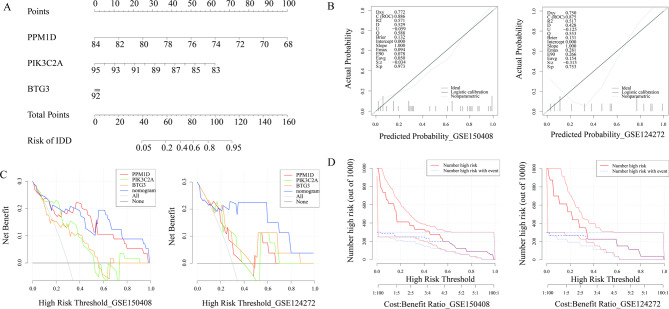
Clinical application of biomarkers. (**A**) Nomogram of three biomarkers. (**B**) Calibration curves for evaluating the predictive performance of the nomogram model in the GSE150408 and GSE124272 datasets. (**C**) DCA curves for assessing the clinical utility of the nomogram model in the two datasets. (**D**) clinical impact curves of the nomogram model in the two datasets.

To facilitate clinical assessment of IDD risk, a nomogram incorporating the three biomarkers was constructed using logistic regression. Each biomarker was assigned a score on its respective subscale within the nomogram; the total score was used to predict the risk of developing IDD, with higher scores indicating a greater risk (Fig. 4A). In the calibration curves of the training set and validation set, the actual risk was highly consistent with the predicted risk from the nomogram model (Fig. 4B). The decision curve analysis (DCA) of the two datasets demonstrated that when the high-risk threshold ranged from 0.00 to 1.00, the clinical benefit of the nomogram model was consistently higher than that of the curves for PPM1D, PIK3C2A, and BTG3 alone (Fig. 4C). The clinical impact curve of the two datasets showed that across the threshold range from 0 to 1, the “Number high risk” curve was closely aligned with the “Number high risk with event” curve under high-risk thresholds (Fig. 4D). In summary, the calibration curve, DCA decision curve, and clinical impact curve collectively confirm that the nomogram exhibits robust predictive accuracy.

### GSEA enrichment analysis of biomarkers

**Fig. 5:**
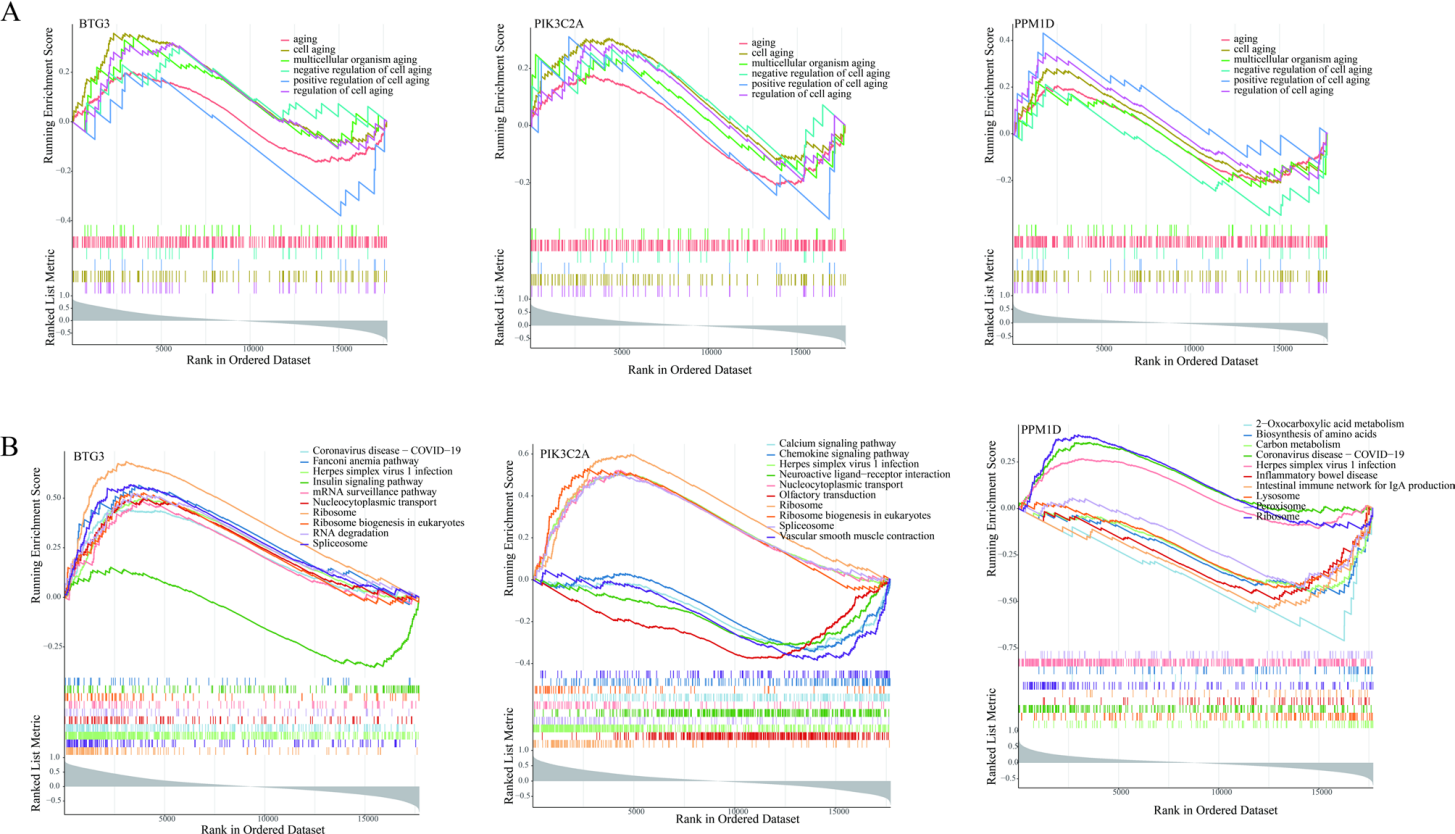
GSEA enrichment analysis of biomarkers. (**A**) GO-Biological Process (GO-BP) enrichment analysis of BTG3, PIK3C2A, and PPM1D. (**B**) KEGG pathway enrichment analysis of BTG3, PIK3C2A, and PPM1D.

To investigate the biological functions of the biomarkers, GSEA was performed in this study. In terms of GO functional annotations, all three biomarkers were enriched in gene functions including aging, cell aging, multicellular organism aging, negative regulation of cell aging, positive regulation of cell aging, and regulation of cell aging (Fig. 5A). Additionally, KEGG pathway analysis results revealed that the three biomarkers were significantly enriched in the “Herpes simplex virus 1 infection” and “Ribosome” pathways (Fig. 5B). These findings suggest that the three biomarkers may influence the progression of IDD through the aforementioned gene functions and metabolic pathways.

### Immune infiltration analysis

**Fig. 6:**
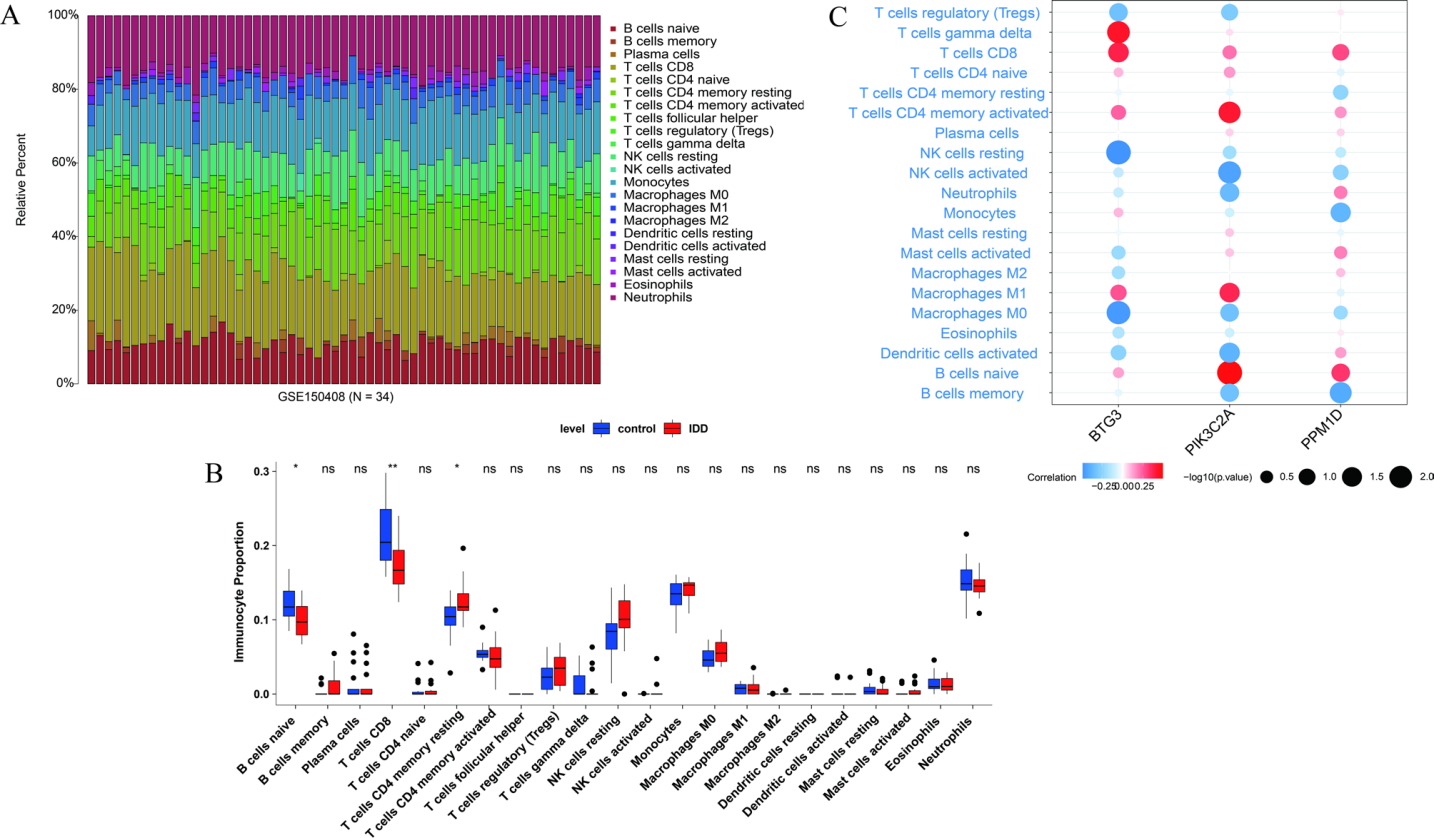
Immune infiltration analysis. (**A**) Stacked bar plot showing the proportion of immune cells. (**B**) Boxplots of immune cell abundance between the IDD and normal groups. (**C**) Correlation heatmap between biomarkers and immune cells.

To investigate differences in the immune infiltration profile of IDD samples, this study employed the CIBERSORT algorithm to quantify the proportions of 22 immune cell types in blood samples from the GSE150408 dataset (Fig. 6A). Specifically, the immune infiltration levels of naive B cells and CD8 + T cells were significantly downregulated in the IDD group, while the infiltration level of resting CD4 + memory T cells was significantly upregulated (Fig. 6B). Additionally, correlation analysis between the biomarkers and immune cells showed that PIK3C2A was significantly positively correlated with activated CD4 + memory T cells and naive B cells (cor > 0.3, *P* < 0.05), whereas BTG3 was significantly negatively correlated with M0 macrophages and resting natural killer (NK) cells (cor < − 0.3, *P* < 0.05) (Fig. 6C).

### Multifactorial biomarker regulatory network and drug prediction analysis

**Fig. 7:**
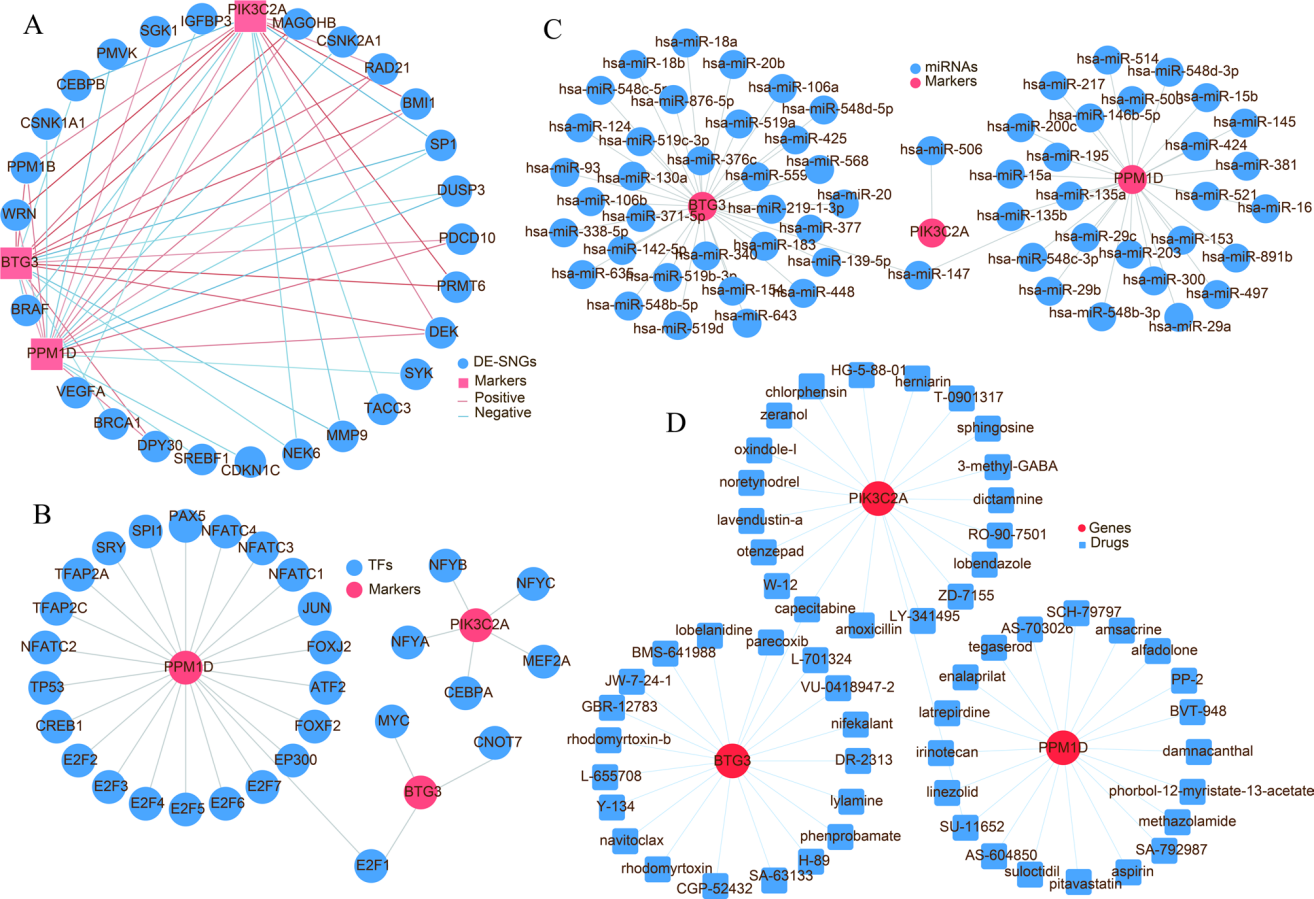
Multifactorial networks of biomarkers. (**A**) CeRNA regulatory network of biomarkers. (**B**) Biomarker-transcription factor (TF) regulatory network. (**C**) miRNA–mRNA regulatory network of biomarkers. (**D**) Drug-biomarker interaction network.

To investigate the relationships between biomarkers, aging-related genes, transcription factors (TFs), and miRNAs, a multi-factor network analysis was conducted in this study. The co-expression network of biomarkers and DESNGs revealed significant interactions between these two groups (Fig. 7A). The biomarker-TF regulatory network identified that the transcription factor E2F1 simultaneously regulates two biomarkers, namely PPM1D and BTG3 (Fig. 7B). The miRNA-mRNA regulatory network uncovered that PPM1D and BTG3 are both regulated by hsa-miR-147 (Fig. 7C). Additionally, the drug prediction network demonstrated that a significant correlation among PPM1D, PIK3C2A, and SU-11,652, as well as a significant correlation among PIK3C2A, BTG3, and capecitabine (Fig. 7D).

### Experimental validation of biomarkers

**Fig. 8:**
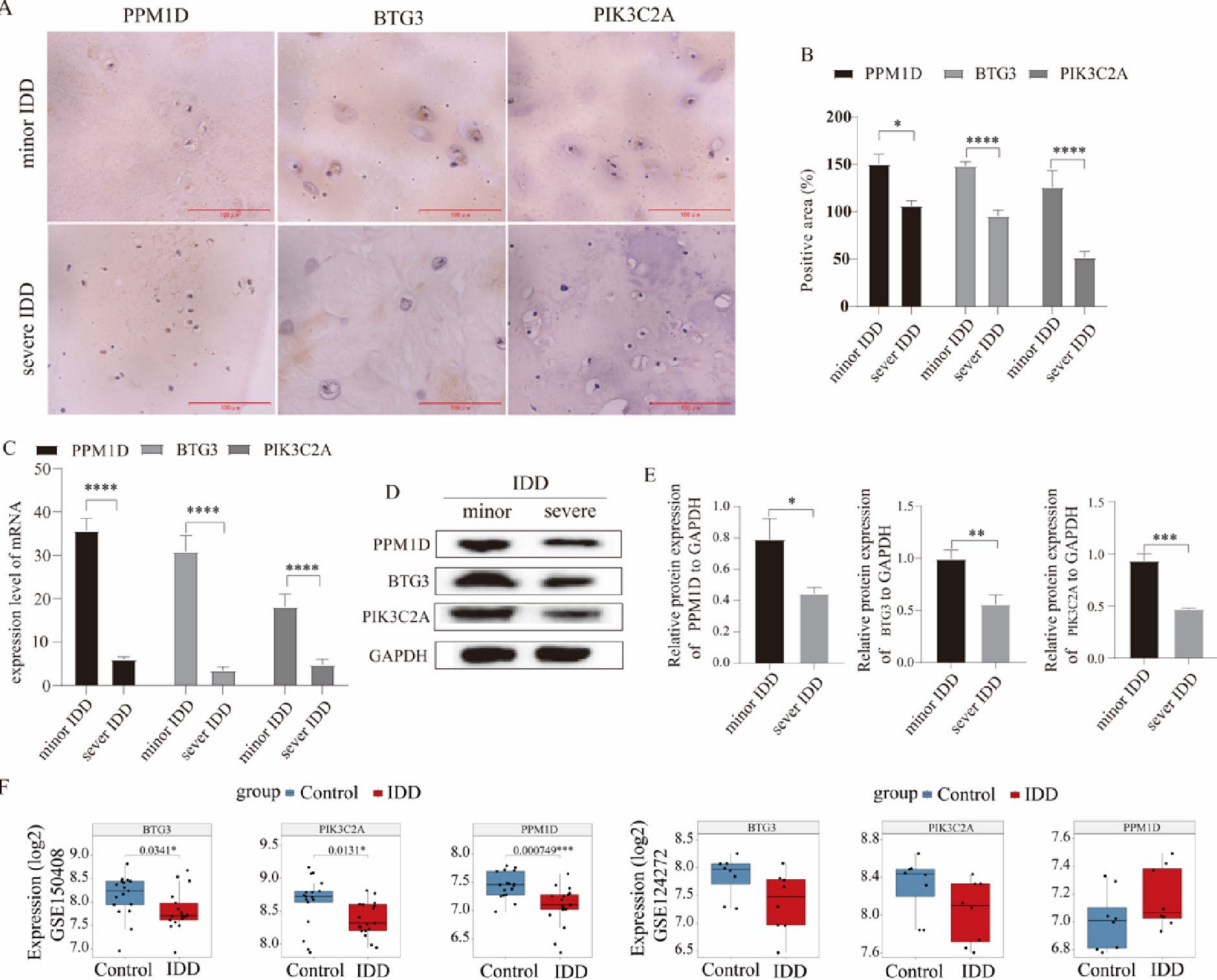
Validation of the expression levels of biomarkers PPM1D, BTG3 and PIK3C2A. (**A**,**B**) Immunohistochemistry (IHC) staining and the corresponding quantitative bar charts; scale bar = 100 μm (**C**) mRNA expression levels of the biomarkers detected by RT-qPCR (**D**,**E**) Protein expression levels of the biomarkers measured by Western blot (WB) and the relevant quantitative bar charts. (**F**) Expression profiles of the biomarkers in both training and validation cohorts, *indicates a statistically significant difference, * ***P < 0.0001.

Based on transcriptome data, three IDD and aging-related biomarkers, namely PPM1D, BTG3 and PIK3C2A, were identified and screened by machine learning in this study. Subsequently, the expression levels of these biomarkers were verified using nucleus pulposus tissues derived from patients with mild and severe IDD. IHC results demonstrated that the expression levels of PPM1D, BTG3 and PIK3C2A were significantly downregulated in patients with severe IDD (Fig. 8A,B). Consistently, RT-qPCR assays revealed a marked downregulation in the expression of these three biomarkers in severe IDD (Fig. 8C, Supplementary Fig. 1). WB analysis also confirmed that the protein levels of the three biomarkers were significantly decreased in severe IDD (Fig. 8D,E). To enhance the persuasiveness of the data, the expression levels of the three biomarkers were extracted separately from both the training and validation cohorts. Combined with the results of validation experiments, The expression levels of BTG3 and PIK3C2A exhibited consistent changing trends in IHC, RT‑qPCR, Western blot analyses, and both datasets, whereas the expression pattern of PPM1D in the validation dataset was completely opposite to those observed in other assays (Fig. 8F). Therefore, BTG3 and PIK3C2A may play more critical roles in the progression of IDD.

## Discussion

Intervertebral disc degeneration (IDD) is a prevalent cause of joint-related chronic disability worldwide, which can induce chronic low back pain and subsequently lead to disability. This condition severely impairs patients’ quality of life and well-being, imposing a substantial public health and economic burden globally^21^. Despite its high incidence, reliable biomarkers for disease diagnosis remain lacking. Studies have revealed accelerated cellular senescence in intervertebral disc degeneration, and the senescent phenotype is associated with increased catabolism. This suggests that cellular senescence plays a role in the pathogenesis of intervertebral disc degeneration^22^. Therefore, in this study, machine learning was employed to identify PPM1D, PIK3C2A, and BTG3 as senescence-related biomarkers in IDD. Notably, PIK3C2A and BTG3 exhibited consistent trends in expression level changes across both datasets and validation experiments, suggesting they may play more critical roles in IDD. This provides a novel reference for the development of innovative therapeutic strategies for patients with IDD.

B cell translocation gene 3 (BTG3) is a member of the B cell translocation gene family. Within the nuclear compartment, BTG3 protein is capable of interacting with multiple molecules including E2F transcription factor 1 (E2F1), receptor-regulated Smad8 transcription factor, and Caf1—a transcriptional cofactor associated with C-C motif chemokine receptor 4 (CCR4)^23^. Collectively, these interactions suppress cell proliferation and cell cycle progression, and induce G0/G1 arrest and apoptosis^24^. In contrast, the senescence and apoptosis of nucleus pulposus cells (NPCs), progressive degradation of the extracellular matrix (ECM), fibrosis of the annulus fibrosus (AF), and inflammatory responses represent the core pathological drivers underlying the development of IDD^25^. These findings suggest that BTG3 may be involved in the progression of IDD by modulating the senescence and apoptosis of NPCs. KEGG analysis demonstrated that the BTG3-related pathways included the insulin signaling pathway, mRNA surveillance pathway, nucleocytoplasmic transport, ribosome biogenesis in eukaryotes, RNA degradation, and spliceosomes. The inhibition of insulin/insulin-like growth factor 1 (IGF-1) signaling can prolong lifespan. However, aging can be promoted when the activity of the insulin/IGF-1 signaling pathway increases. Moreover, the insulin/IGF-1 pathway plays an important role in the synthesis of proteins, energy metabolism, and proliferation and differentiation of insulin/IGF-1-responsive cells^26^. This suggests that BTG3 may affect cell proliferation and differentiation via the insulin signaling pathway, thereby influencing the progression of IDD. However, the specific underlying molecular mechanisms remain to be further explored.

PIK3C2A is identified as a class II member of the phosphoinositide 3-kinase family and binds to an aptamer protein via a proline-rich region present in the N-terminal sequence^27^. A reduction in PI3K-C2A expression is linked to senescence^28^. Liu et al.^29^ demonstrated that PIK3C2A overexpression could enhance cell viability, reduce apoptosis, and inhibit the secretion of inflammatory cytokines. Given that the apoptosis and inflammation of NPCs are known to exacerbate the progression of IDD, the downregulated expression of PIK3C2A may, to a certain extent, indicate the aggravation of IDD. In the present study, the results of IHC and RT-qPCR showed that the expression level of PIK3C2A was significantly decreased in severe IDD compared with that in mild IDD, which was consistent with the findings of previous studies. KEGG analysis demonstrated that PIK3C2A-related pathways included Olfactory transduction, Chemokine signaling and Spliceosome. Chemokines are a group of small secreted molecules that signal through G-protein-coupled receptors, promoting cell survival and proliferation while providing directional guidance for migrating cells^30^. This suggests that PIK3C2A may influence cellular senescence and apoptosis via chemokine signaling, thereby participating in the progression of IDD.

PPM1D is a protein phosphatase belonging to the PP2C family of serine/threonine protein phosphatases. This protein phosphatase type negatively regulates the cellular responses to stress^31^. PPM1D encodes a serine/threonine phosphatase termed WIP1, which acts as a negative regulator of p53 activity and also functions as a key protein involved in cell cycle regulation, DNA repair, and apoptosis^32^. DNA damage is a physiological event that occurs constantly in the human body, and the accumulation of such damage may induce cellular senescence and apoptosis^33^. Cells are equipped with an intricate DNA repair system to counteract DNA damage, which can alleviate the progression of IDD to a certain extent. Taken together, these findings suggest that PPM1D may influence the progression of IDD by participating in cellular DNA repair processes. However, the specific underlying molecular mechanisms remain to be further explored. KEGG analysis demonstrated that the PPM1D-related pathways included carbon metabolism, intestinal immune network for IgA production, lysosomes, and peroxisomes. Lysosomes play an indispensable role in diverse types of autophagy, including micro-autophagy, macro-autophagy, and chaperone-mediated autophagy, as well as in multiple cellular death pathways^34^. Lysosomal impairment may induce lysosomal membrane permeabilization and trigger various forms of cell death, including apoptosis. Apoptosis is closely associated with cellular senescence, and the apoptosis and senescence of NPCs are well recognized as critical pathological contributors to IDD. Therefore, PPM1D may be involved in the progression of IDD via the lysosomal pathway. However, their specific gene functions in IDD still require further in-depth experimental validation.

GSEA enrichment analysis revealed that all three biomarkers were significantly enriched in the “Herpes simplex virus 1 infection” and “Ribosome” pathways. The ribosome is a multi-subunit complex responsible for translating mRNA into protein, and ribosome biogenesis, defined as the de novo synthesis of ribosomes, plays critical roles in multiple cellular processes including cell proliferation, differentiation, apoptosis, development, and transformation^35^. Previous studies have demonstrated that cellular senescence is accelerated in IDD, and the senescence phenotype is closely associated with the pathogenesis of IDD. These findings suggest that PPM1D, PIK3C2A, and BTG3 may participate in the initiation and progression of IDD via the ribosome pathway, although their detailed molecular mechanisms warrant further investigation.

Immune infiltration analysis revealed that PIK3C2A was significantly positively correlated with activated CD4 + memory T cells and naive B cells, while BTG3 exhibited a significant negative correlation with M0 macrophages and resting natural killer (NK) cells. Studies have indicated that CD4 + T cells can affect the function of intervertebral disc cells and the metabolism of extracellular matrix by secreting cytokines, thereby promoting intervertebral disc degeneration^36^. Additionally, *Qin et al.*^37^. identified a significant causal relationship between naive B cells and IDD through MR studies. These findings suggest that PIK3C2A may be involved in immune responses via CD4 + memory T cells and naive B cells, thereby influencing the progression of IDD. Furthermore, excessive activation of NF-κB promotes inflammatory responses and exacerbates the degenerative changes of the intervertebral disc, while the migration number of inactivated macrophages (M0) increases significantly in an inflammatory environment^38^. This implies that BTG3 may participate in the immune response of IDD through M0 macrophages.

Through bioinformatics analysis, our study initially identified three aging-related biomarkers associated with IDD, namely PPM1D, PIK3C2A, and BTG3. This finding provides novel insights for the development of innovative therapeutic strategies for IDD. However, this study has certain limitations. For instance, the sample sizes of both the training and validation cohorts are relatively small, which are confounded by age-related factors. Furthermore, blood transcriptome data was used as surrogate data in this study, which may have exerted a certain impact on the results. Considering this confounding factor, more relevant clinical nucleus pulposus samples were employed for biomarker validation in our study. Detection in nucleus pulposus tissues revealed that the expression trends of the biomarkers PIK3C2A, and BTG3 in IHC, RT-qPCR and WB assays were consistent with those observed in the datasets (both validation and training sets). These two biomarkers may play a more critical role in IDD. This has to a certain extent compensated for the limitations of using blood-derived data as a surrogate dataset. In future studies, we will further expand the sample size and optimize the sample collection strategy. We will validate the expression levels of the identified biomarkers in patient blood and explore the correlation of biomarker expression between blood and IDD tissue samples using correlation analysis. In addition, we will complement relevant in vitro and in vivo experiments to further validate the gene functions of the identified biomarkers in IDD.

## Conclusion

LASSO, SVM-RFE, and ROC analyses identified PPM1D, PIK3C2A, and BTG3 as aging-related biomarkers in IDD. Notably, PIK3C2A and BTG3 exhibited consistent expression trends across transcriptome datasets, IHC, RT-qPCR and WB assays, suggesting that they may play more critical roles in intervertebral disc degeneration (IDD). These findings provide novel insights for molecular investigations into the progression of IDD.

## Supplementary Information

Below is the link to the electronic supplementary material.

Supplementary Material 1

Supplementary Material 2

Supplementary Material 3

## Data Availability

All data in this study are available from the GEO database. The datasets and their corresponding direct access links are as follows: GSE150408 (https://www.ncbi.nlm.nih.gov/geo/download/?acc=GSE150408&format=file) and GSE124272 (https://www.ncbi.nlm.nih.gov/geo/download/?acc=GSE124272&format=file).
