# Supplementary figures and images for: Identification and experimental validation of aging-related biomarkers in intervertebral disc degeneration
Source: Sci Rep. 2026 Apr 9;16:16716. doi: 10.1038/s41598-026-47889-6 (PMC13222862; doi:10.1038/s41598-026-47889-6)

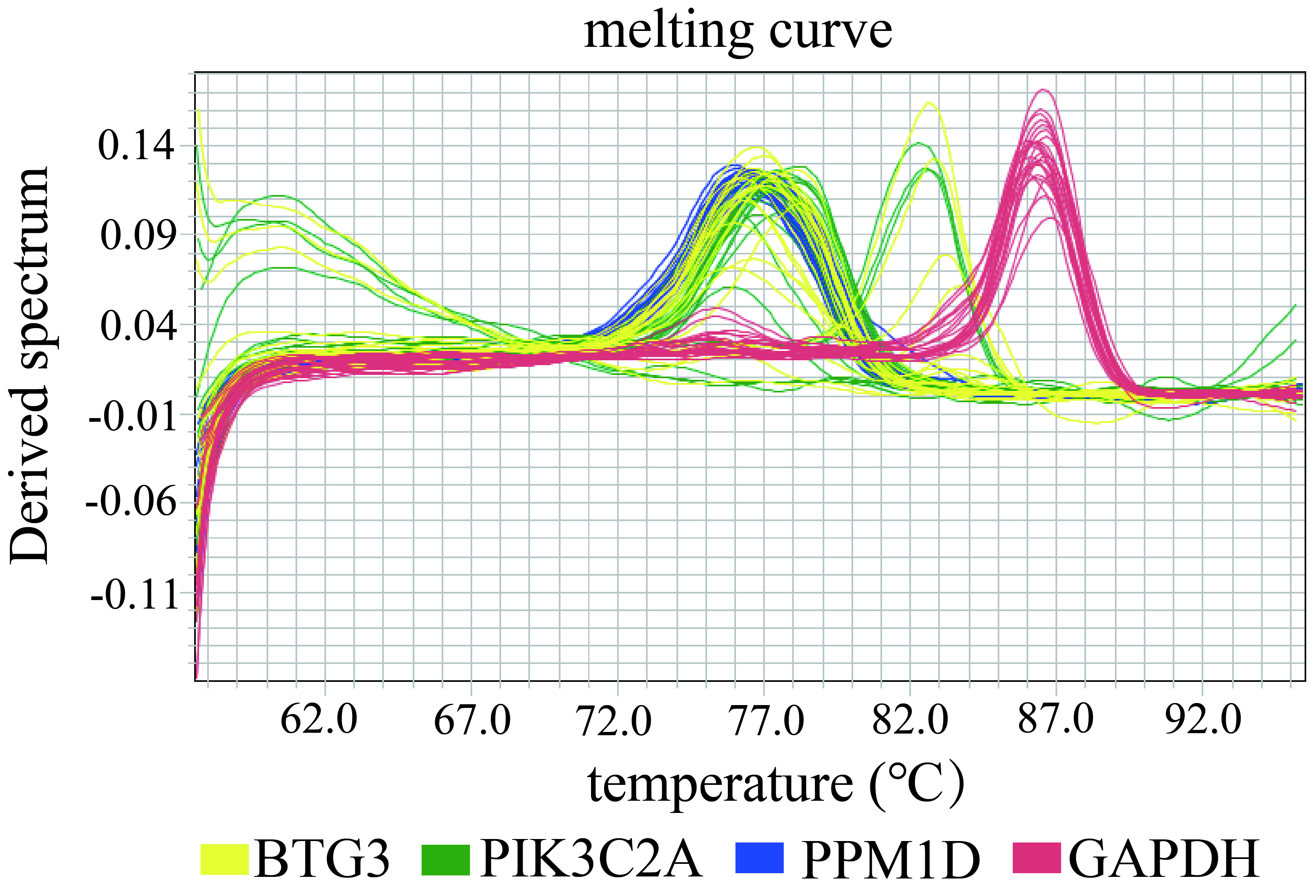

Supplement: Supplementary file 2 — Supplementary Material 2 [file 41598_2026_47889_MOESM2_ESM.jpg]

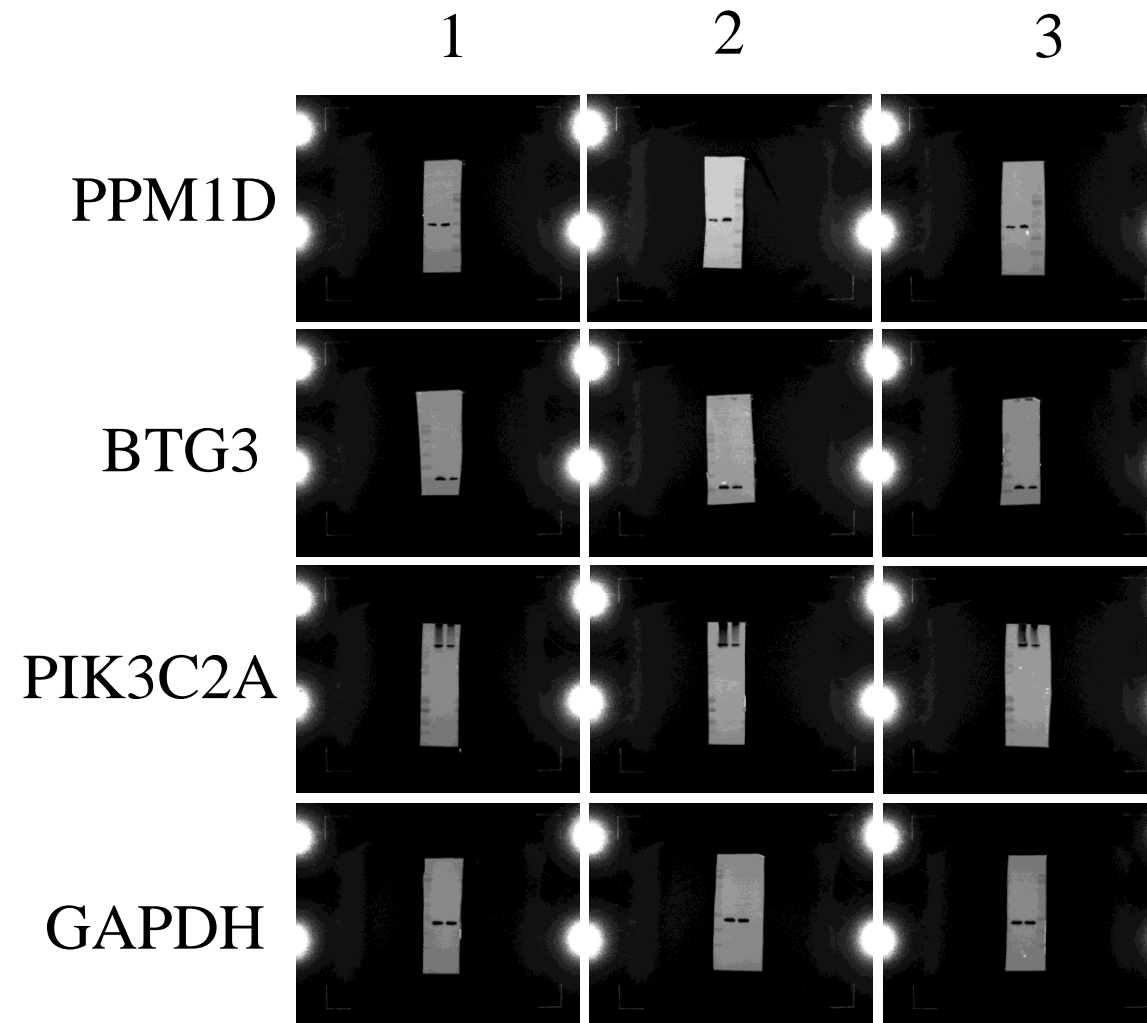

Supplement: Supplementary file 3 — Supplementary Material 3 [file 41598_2026_47889_MOESM3_ESM.pdf]
